# Simple Webserver-Facilitated Method to Design and Synthesize Artificial miRNA Gene and Its Application in Engineering Viral Resistance

**DOI:** 10.3390/plants11162125

**Published:** 2022-08-15

**Authors:** Muhammad Yasir, Mohamed Motawaa, Qingwei Wang, Xi Zhang, Annum Khalid, Xingkui Cai, Feng Li

**Affiliations:** 1 Key Laboratory of Horticultural Plant Biology (MOE), College of Horticulture and Forestry Sciences, Huazhong Agricultural University, Wuhan 430070, China; 2 Agricultural Microbiology Department, Agriculture Faculty, Damietta University, Damietta 34511, Egypt

**Keywords:** artificial miRNA, gene synthesis, crop protection, potato leafroll virus, grafting

## Abstract

Plant viruses impose serious threats on crop production. Artificial miRNAs can mediate specific and effective gene silencing in plants and are widely used in plant gene function studies and to engineer plant viral resistance. To facilitate the design of artificial miRNA genes, we developed a webserver, AMIRdesigner, which can be used to design oligos for artificial miRNA synthesis using wild-type and permutated MIR171 and MIR164 backbones. The artificial miRNA genes designed by AMIRdesigner can be easily assembled into miRNA clusters for multiple target sites. To validate the server functionality, we designed four artificial miRNA genes targeting four conserved regions in the potato leafroll virus genome using AMIRdesigner. These genes were synthesized with the server-designed oligos and further assembled into a quadruple miRNA cluster, which was cloned into an overexpression vector and transformed into potato plants. Small RNA Northern blot and virus inoculation analyses showed that a high level of artificial miRNA expression and good viral resistance were achieved in some of the transgenic lines. These results demonstrate the utility of our webserver AMIRdesigner for engineering crop viral resistance.

## 1. Introduction

Small RNAs, including microRNA (miRNA) and small interfering RNAs (siRNA), are short non-coding RNAs 20 to 24 nucleotides (nts) long that direct sequence-specific gene silencing in complex with various plant argonaute (AGO) proteins through mRNA degradation, translation inhibition or transcription repression [1,2,3]. miRNA and siRNA have similar chemical properties and function mechanisms but differ in their precursors. Plant miRNAs are encoded by endogenous long non-coding RNAs (lncRNA) with relatively short fold-back structures, which are called primary miRNA precursors (pri-miRNAs). In plants, pri-miRNAs are mainly processed by Dicer-like 1 (DCL1) enzyme into short miRNA/miRNA* duplexes through two or more successive cleavage reactions. The short duplex can be methylated at the 2′ hydroxyl group of the 3′ end nucleotide by Hua Enhancer 1 (HEN1) and then associated with AGO1 to form mature RNA-induced silencing complex (RISC) with the miRNA* degraded [2,4]. Precursors of siRNA come from a variety of sources, including double-stranded RNA from plant virus replication intermediates, plant RNA-dependent RNA polymerase (RDR) and long inverted repeated RNAs [5].

In plants, viral replication results in the formation of a double-stranded RNA intermediate, which is processed by DCL2 and DCL4 into 22 and 21 nucleotide (nt) siRNAs [6,7]. The viral siRNAs are loaded into AGO1 and AGO2 to form viral siRNA programed RISC, which represses viral gene expression based on complementarity between viral siRNA and viral transcripts and plays an important role in antiviral defense [8,9]. This defense mechanism is called antiviral RNA silencing [10,11]. In the natural antiviral RNA silencing process, viral siRNA is produced after viral replication or gene expression. Many viruses encode suppressors of RNA silencing that can inhibit antiviral RNA silencing using many different mechanisms, thus helping viruses to overcome the viral siRNA-mediated defense and cause disease symptoms [12,13,14].

The discovery of plant small RNAs was paved by the efforts toward engineering plant resistance to viruses, and mechanistic dissection of RNA silencing has, in turn, greatly facilitated applications involving the engineering of small RNA-based viral resistance [15]. Due to their well-understood biogenesis mechanism and precision in processing, different miRNA precursors have been employed as backbones to replace mature miRNA/miRNA* sequences with sequences of interest and generate artificial miRNAs. These can be used to mediate specific and efficient silencing of target genes in plants and have been applied in engineering plant viral resistance [16,17]. In transgenic plants expressing artificial miRNA targeting viral transcripts, amiRNA-programed RISCs form in the absence of viral replication and gene expression. They may thus cleave viral transcripts as soon as they are released in plant cells and mediate effective antiviral defense in conjunction with viral siRNA-programed RISCs.

Plant viruses cause serious problems in crop production, and mixed infections by many viruses usually occur on different crops [15,18]. In practice, there is a need to express multiple artificial miRNAs that target multiple viruses, to cope with mixed virus infections, or multiple viral genes from the same virus, to ensure strong resistance against the targeted virus. To facilitate applications of artificial miRNAs in crop protection, we developed a webserver, AMIRdesigner, that provides multiple artificial miRNA backbones derived from highly expressed plant miRNAs and can be used to design primer sets for synthesis of artificial miRNA constructs based on input mature miRNA sequences. For proof of concept, we selected four artificial miRNA sequences targeting conserved regions from potato leafroll virus (PLRV). A quadruple miRNA cluster expression vector was constructed using the primers designed in the webserver and transformed into potato plants. Two potato transgenic lines expressing high-level artificial miRNAs and conferring good resistance against PLRV were selected after PLRV inoculation. This study provides a simple and fast method to design and synthesize artificial miRNA constructs for the engineering of viral resistance, as well as for the silencing of specific plant genes. AMIRdesigner is available at http://lifenglab.hzau.edu.cn/Tools/index.php?tid=AMIRdesigner (accessed on 10 June 2022).

## 2. Result

### 2.1. AMIRdesigner, a Webserver for Designing Primary Artificial miRNA Precursor Sequences

For the expression of an artificial miRNA of interest, we chose two highly expressed miRNA with relatively short primary sequences, pri-miR171 and pri-miR164, as the backbone [19]. Three versions of the miR171 and miR164 backbone were created, one with the wild-type lower stem and upper loop, the other two with mutated sequences but the same secondary structures in the lower stem and upper loop (Figure S1). The sequence variation in the different backbones makes the fusion PCR step more efficient when multiple artificial miRNA precursors need to be clustered for various purposes. To synthesize the artificial miRNA primary transcript coding sequence, five oligo nucleotide sequences were designed (P1, P2, P3, P4 and P5; Figure S1). Among those oligos, P1, P3 and P5 were common primers specific to each backbone sequence, while P2 and P4 were specific to the mature artificial miRNA of interest and backbone sequences.

**Figure 1:**
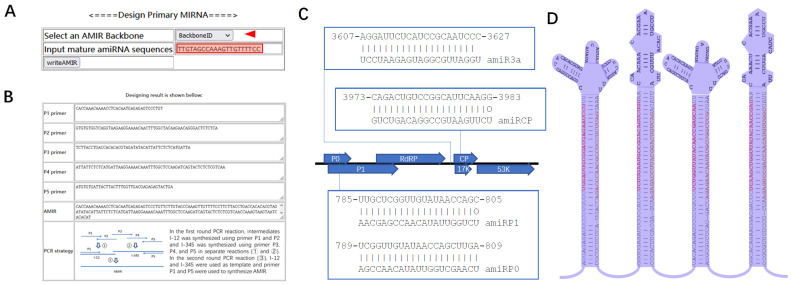
Interface of AMIRdesigner. (**A**) Homepage of AMIRdesigner. The red arrowhead points to the pulldown menu where users can select the backbone of the artificial miRNA; the textbox highlighted in red is where users can input their designed mature miRNA sequences of 21 or 22 nts in length. (**B**) Output from AMIRdesigner. Sequences of common primers specific to each backbone (P1, P3 and P5) and primers specific to each mature artificial miRNA (P2 and P4), as well as designed primary artificial miRNA (AMIR), are shown on the top rows. The PCR strategy using the designed primers to synthesize the designed AMIR is illustrated at the bottom. (**C**) AMIR target sites in the PLRV genome. The reference genome of D00530.1 was used. ”|” indicates a matched base while ”o” indicates a U-G wobble pair. 5′ U was used in all mature amiRNAs to facilitate their loading into the AGO1 protein. (**D**) Secondary structure of the designed PLRV targeting the AMIR cluster. Mature miRNA sequences are in red and bolded while miRNA* sequences are in dark yellow.

To simplify the design process for the primary artificial miRNA sequences, a webserver, AMIRdesigner, was set up. On the homepage of the webserver, users can select an miRNA backbone from a pulldown menu and paste the mature artificial miRNA of interest (Figure 1A). After clicking the “writeAMIR” button, the five oligo sequences and the designed primary miRNA coding sequences will be printed and a fusion PCR pipeline to generate the designed primary miRNA coding sequences will be shown (Figure 1B).

### 2.2. Design and Synthesis of Multiple AMIRs Targeting Potato Leafroll Virus

To test the functionality of AMIRdesigner, we designed an AMIR targeting potato leafroll virus to test its resistance to PLRV in potato. For this, four conserved target sites in the PLRV genome were selected (Figure 1C) and backbone AMIR171v1, AMIR164v1, AMIR171v2 and AMIR164v2 were chosen to design AMIR3a, AMIRCP, AMIRP1 and AMIRP0, respectively. These four AMIRs were further linked together to form a four-miRNA cluster (Figure 1D).

**Figure 2:**
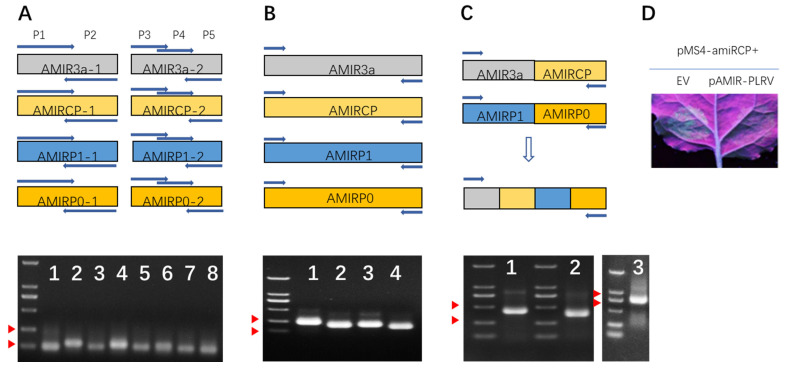
Synthesis of AMIR unit and cluster using fusion PCR protocol. (**A**) top, structure of subunits 1 and 2 of each AMIR, arrows on top of each subunit represent primers 1 to 5 (not drawn to scale); bottom, agarose gel image of the first-round PCR products, red arrowheads point to DNA ladders of 100 and 250 nt, respectively. (**B**) Structure and product of second-round PCR resulting in four individual AMIRs. Red arrowheads point to DNA ladders of 250 and 500 nt, respectively. (**C**) The third- and fourth-round PCR, producing double and quadruple AMIR clusters. Red arrowheads point to DNA ladders of 500 and 750 nt, respectively. (**D**) Transient assay in Nicotiana benthamiana leaf, photo taken at 3 day post-agrobacterium infiltration under UV light. Red arrowheads point to DNA ladders of 750 and 1000 nt, respectively.

AMIR synthesis was completed in a two-round PCR reaction. In the first-round PCR, part 1 and part 2 of the fragments of each AMIR were synthesized using partially overlapping P1-P2 and P3-P4-P5 primers, respectively (Figure 2A). In the second-round PCR, part 1 and part 2 were used as templates, and P1-P5 primers were used to amplify the fusion product (Figure 2B). As 18 overlapping nucleotide sequences were designed in the P1 and P5 primers of the adjacent AMIRs (Figure 1C, Table S1), in a third-round PCR reaction, dual AMIR clusters AMIR3a–AMIRCP and AMIRP1–AMIRP0, were synthesized. These two dual clusters were further assembled into a final quadruple AMIR cluster using fusion PCR, taking advantage of the designed overlapping sequences between AMIRCP and AMIRP1 (Figure 2C, Table S1). Finally, the AMIR cluster was fused together with a fragment of a GUS sequence and cloned into a binary vector using restriction digestion and ligation to produce the pAMIR–PLRV vector (Supplementary Data S1).

To validate the expression and function of the artificial miRNA from pAMIR–PLRV, a miRNA sensor construct pMS4–amiRCP was constructed, which had a binding site for amiRCP (Table S2). pAMIR–PLRV and pMS4–amiRCP were both transformed into Agrobacterium GV3101. Agrobacteria harboring pAMIR–PLRV and pMS4–amiRCP, as well as agrobacteria harboring an empty vector (EV) and pMS4–amiRCP, were infiltrated into N. benthamiana leaves. When co-infiltrated with EV agrobacterium, pMS4–amiRCP T-DNA was transferred into the plant cells and directed transcription of GFP mRNA, which has a amiRCP binding site. As the EV did not directly produce any miRNA, GFP mRNA was stable and translated into GFP protein. Thus, the infiltrated patch of N. benthamiana leaves showed a green florescence signal under UV light (Figure 2D, left). In contrast, when co-infiltrated with pAMIR–PLRV agrobacterium, pAMIR–PLRV T-DNA directed transcription of the AMIR–PLRV miRNA cluster, which was processed into four mature artificial miRNAs, including amiRCP (Figure 1C), and incorporated into AGO1 to form RISC. Although, in this case, pMS4–amiRCP T-DNA directed a similar level of transcription of GFP mRNA, amiRCP-programed RISC could bind to the mRNA at its amiRCP binding site and direct cleavage and translation inhibition of GFP mRNA. As a result, less GFP protein was accumulated and a weaker green florescence signal was observed in the pMS4–amiRCP and pAMIR–PLRV agrobacteria co-infiltrated patch compared with the EV and pMS4–amiRCP agrobacteria co-infiltrated patch (Figure 2D, right). This result indicated that the AMIR cluster expression construct was functional.

### 2.3. AMIR–PLRV Directs High-Level amiRNA Accumulation and Confers Resistance to PLRV in Transgenic Potato

**Figure 3:**
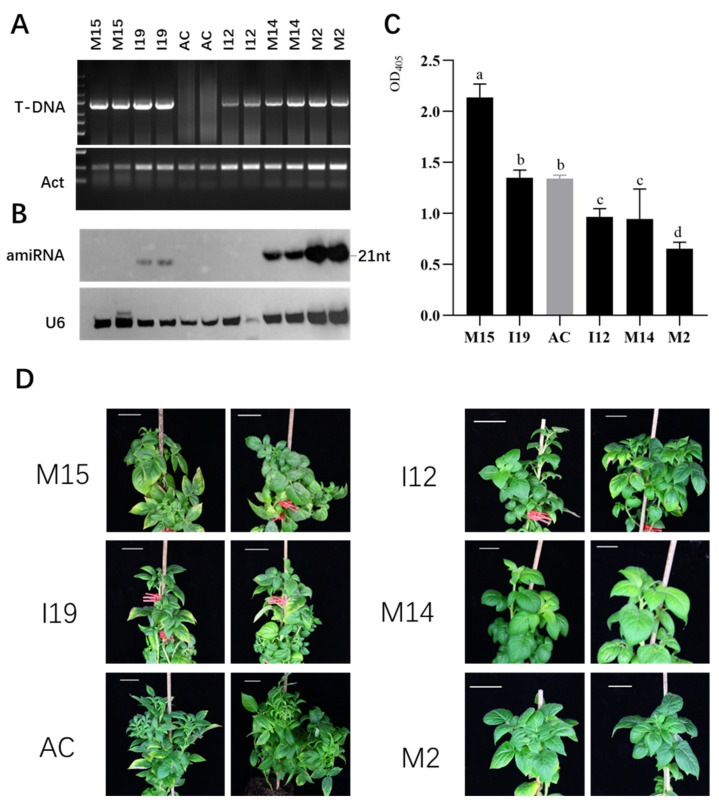
Characterization of pAMIR–PLRV transgenic plants. (**A**) PCR detection of pAMIR–PLRV T-DNA insertion in transgenic and wild-type potato lines. Transgenic line IDs (M15, I19, I12, M14 and M2) and wild-type control lines AC142 (AC) are indicated at the top of each lane. PCR products amplified using pAMIR–PLRV T-DNA and endogenous control actin DNA (Act) are indicated to the left of each gel. (**B**) Northern blot detection of mature miRNAs expressed from PLRV–AMIR. Mixed probes complementary to all four amiRNAs were used. The blot of U6 RNA served as an equal loading control. amiRNA size is indicated to the right of the blot. (**C**) ELISA assay for samples from top young leaves of infected plants using PLRV CP-specific antibody. The difference among the ELISA values of different samples was analyzed by Duncan’s new multiple range test and was indicated by letters above each column. (**D**) Disease symptoms in wild-type (AC142) and transgenic potato lines (M15, I12, I19, M14, M2) after PLRV infection.

To test its function in mediating resistance against PLRV in potato, pAMIR–PLRV was transformed into potato diploid cultivar AC142. Several lines of positive transformants were obtained and clonally propagated. After the initial PCR test, five T-DNA insertion positive lines were selected for small RNA detection (Figure 3A). The results showed that accumulation levels of amiRNAs in different lines varied significantly and lines M14 and M2 showed higher accumulation levels, while the U6 RNA loading control was detected in all samples with a strong signal (Figure 3B).

Next, the reaction of transgenic potato plants to PLRV was tested using the graft inoculation method. For this, plants grown in soil were topped off and a leaf from the PLRV reservoir potato plants was grafted onto the root stock from transgenic plants and wild-type controls (Figure S2). Sixty-five days after grafting, when there was still no clear viral symptom, the top young leaves from the new shoots of the rootstock were harvested for the ELISA test. The results showed absorbance values of around 1.3 or above for lines M15 and I19 and the wild-type control AC142, while about 0.9 and 0.6 were observed in I12, M14 and M2, which were lower than the control and other lines (Figure 3C). In our experience, absorbance values of 0.3 or below indicate no infection. These ELISA results suggested that M14 and M2 accumulated low levels of PLRV. Since, during graft inoculation, large amounts of virus are loaded directly into the phloem system, which may provide a much higher infection pressure compared to the virus load during natural infection through aphis, we expected that lines M14 and M2 would achieve better resistance in natural infection. At day 70 after grafting, clear leafroll symptoms appeared in the wild-type control and lines M15 and I19 and mild symptoms appeared in line I12, while the leaves in lines M14 and M2 remained healthy (Figure 3D). These results showed that transgene AMIR–PLRV conferred good resistance to PLRV in transgenic lines M14 and M2, which is consistent with the high level of amiR–PLRV accumulation.

## 3. Discussion

Artificial miRNAs are useful tools for the silencing of specific endogenous genes and for engineering viral resistance in plants. In this study, we developed a webserver, AMIRdesigner, to facilitate applications of artificial miRNA in these areas. Previously, artificial miRNA precursor was synthesized using three pair of primers and, as a template DNA containing the backbone miRNA gene was needed, one pair of primers were backbone-specific and two pairs of primers were specific to the desired mature miRNAs and backbone [16]. Synthesis of artificial miRNA genes designed in AMIRdesigner does not require a template DNA. Three backbone-specific primers and two amiRNA-specific primers overlapping with each other were assembled into the designed precursor sequences in two rounds of a fusion PCR reaction (Figure 1B and Figure S1). Backbone-specific primers can be reused when different AMIR genes are synthesized, which is economically efficient when high-throughput AMIR gene synthesis is conducted. In plants, when there is an asymmetric bulge in the mature miRNA strand of the miRNA/miRNA* duplex, DCL1 cleavage of the precursor generates 22 nt miRNA instead of the regular 21 nt miRNAs [20]. The 22 nt miRNA-programed AGO1 RISC can trigger phased siRNA synthesis from a cleaved target transcript, which can potentiate miRNA-mediated silencing of endogenous genes [21,22,23]. Besides designing regular 21 nt mature miRNA-producing AMIR precursors, AMIRdesigner can check the length of input mature miRNA sequences. If the length of the input sequences is 22 nt, AMIRdesigner will delete one nucleotide in the miRNA* region of the P4 primer, so that an asymmetric bulge will be created in the miRNA/miRNA* region to produce 22 nt mature miRNAs. This function will enhance its application for the silencing of plant endogenous genes and is not provided by previously published artificial miRNA design webservers, such as Web-based miRNA Designer (WMD) [24] and the Plant Small RNA Maker Site (P-SAMS) [25].

AMIRdesigner provided six AMIR backbones derived from MIR171 and MIR164, which are highly expressed in plants. We designed four AMIRs targeting different genes from PLRV using four different backbones, and the resulting AMIR sequences were efficiently assembled into one cluster using fusion PCR (Figure 2), providing an easy approach to build multiple AMIR clusters. The potato transformation and virus inoculation assay showed that these AMIRs designed in AMIRdesigner functioned well in mediating PLRV resistance (Figure 3), which validated the functionality of our webserver in designing artificial miRNA genes.

In summary, our study provides a simple method for rapid synthesis of artificial miRNA genes, which will promote applications of artificial miRNA in crop protection and plant gene function studies.

## 4. Materials and Methods

### 4.1. Vector Construction

Four AMIRs of PLRV were fused together to construct a single vector AMIR–PLRV. For this purpose, four rounds of fusion PCR amplification were undertaken. The first and second rounds of PCR amplification included initial denaturation for 2 min at 94 °C, followed by 25 cycles of denaturation at 94 °C for 30 s, annealing at 55 °C for 30 s, extension at 72 °C for 1 min and a final extension cycle at 72 °C for 2 min. The third and fourth rounds included initial denaturation for 2 min at 94 °C, followed by 30 cycles of denaturation at 94 °C for 30 s, annealing at 58 °C for 30 s, extension at 72 °C for 1 min and a final extension cycle at 72 °C for 3 min.

For pAMIR–PLRV construction, the PCR product was digested with SpeI restriction enzyme, and 35S promoter-driven overexpression vector pK7LIC1.0 (Figure S3) was digested with SpeI and SmaI restriction enzymes. The digested PCR product and vector were ligated using T4 DNA ligase and transformed into Escherichia coli (*E. coli*) DH5α. To produce miRNA sensor construct, two oligos bearing an amiRCP binding site were annealed together, forming a duplex DNA structure with “TCGA” and “CTAG” 5′ overhangs at each end. This structure was then ligated into the pMS4 vector [26] digested with XhoI and XbaI, which produced sticky ends compatible with the annealed duplex DNA. The ligation reaction was transformed into *E. coli* DH5α.

### 4.2. Potato Transformation

Artificial miRNA expression vector pAMIR–PLRV was transformed into potato cultivar AC142, as described previously [27]. The plantlets were propagated by subculturing of the node cuttings in vitro on MS medium every 4 weeks. The single-node cuttings containing one leaf were shifted into microtuber initiation medium. Microtubers were harvested 16 weeks later, washed in tap water, air-dried at room temperature and then stored in the dark. Upon transformation, microtubers were sectioned into discs 1–3 mm thick. Care was taken to remove any buds from the discs. Then, the discs were submerged in Agrobacterium tumefaciens GV3101 harboring the pAMIR–PLRV vector of the optical density (at 600 nm) of 0.5~1.0 for 10 min and shaken gently every 3 min. Next, the discs were blotted on sterile filter paper to remove excess agrobacterium and placed in co-cultivation medium (3% sucrose M.S. with 0.5 mg/L 6-benzylaminopurine (6-BA), 0.2 mg/L indolacetic acid (IAA) and 0.2 mg/L gibberellic acid (GAӡ)) for 2 days. After co-cultivation, the discs were transferred into P2 medium (3% sucrose M.S. with 0.5 mg/L 6-BA, 0.2 mg/L IAA, 0.2 mg/L GAӡ, 2 mg/L zeatin (ZT), 400 mg/L cefotaxime (Cef) and 75 mg/L kanamycin (Kan)), which was replaced with fresh medium every 2–3 weeks. After about 4–5 weeks, when the regenerated shoots from the microtuber discs reached about 0.5~1.0 cm, they were transferred to magenta boxes containing P3 medium (3% sucrose M.S. supplemented with 50 mg/L Kan. and 200 mg/L Cef.) until roots appeared after about 10 days. Transgenic potato plants cv. AC142 (T0 generation) were maintained in a growth room for virus inoculation bioassays. For this purpose, 13 independent lines (M2, I4, I12, M14, M15, I19, I20, I21, I23, I24, I26, I25 and I27) of in vitro grown transgenic plants and 1 line of untransformed wild-type control plants (cv. AC142) were gardened in plastic pots (clay/sand/peat-moss growth medium). Ten (10) plants (replicates) of each transgenic line, as well as the control lines, were raised.

### 4.3. Virus Infection by Grafting

For graft inoculation, scions (individual leaf or pinnule) were excised from symptomatic parts of the virus infected plant. Test plants were used as root stock. Before grafting, the in vitro propagated wild-type and transgenic potato cultivar AC142 plants were transferred into soil and covered with a transparent plastic cup for 2~3 days (Figure S2). About 38 days later, when the seedlings grew stronger, inoculation was undertaken by cleft grafting. The graft insertion side was covered tightly with parafilm to prevent withering and the plant was further strengthened with a grafting clip and wooden sticks. The grafted plant was protected from excessive evaporation by a plastic bag cover. This cover was removed after 4–5 days (establishment of infection in the recipient plant was achieved when the graft partners had reached a vascular connection for only a short period).

### 4.4. Transient Assay by Agroinfiltration

The transient assay was performed in N. benthamiana as described previously [26]. Briefly, the wild-type tobacco plants were grown in a glasshouse maintained under constant conditions of 60% relative humidity with a day/night photoperiod of 16 h light at 25 °C followed by 8 h dark at 20 °C, and 4~5 week old seedlings (from germination) were used for inoculation. Agrobacterium strains harboring the expression plasmids were cultured in LB medium with proper antibiotics at 28 °C for about two days to reach saturation. Then, the saturated bacterium culture was 50 times diluted in fresh LB medium containing 10 mM MES pH = 5.6 and 20 μM acetosyringone and grown for about 16 h. Cultures were centrifuged at room temperature with 3500 rpm to pellet the bacteria, then resuspended in the infiltration medium containing 10 mM MgCl2 and 150 μM acetosyringone. The optical density of the cultures was determined and diluted to OD = 0.1. Culture was incubated in the infiltration medium for 1–3 h at room temperature before infiltration. The agrobacterium culture of pMS4–amiRCP was co-inoculated with empty vector and pAMIR–PLRV using the pressure infiltration method. GFP expression signals were monitored at 3 and 4 days post-infiltration under a 365 nm UV lamp.

### 4.5. ELISA Assay

The PLRV ELISA test was conducted as described previously [28]. Briefly, coating antibody was diluted using a 1:200 ratio with coating buffer (carbonate buffer, pH = 9.6) and added to a 96-well plate with 100 μL per well. The plate was incubated in a 37 ℃ incubator for 4 h. After incubation, buffer in the plate was removed and the plate was washed three times using 200 μL PBST per well. The leaf sample was ground into leaf sap using a grinding machine and about 200 μL leaf sap was diluted in 800 μL extraction buffer. Then, 100 μL of diluted supernatant was added to the prepared plate with negative and positive controls and an extraction buffer control. The plate was incubated at 4 ℃ overnight and then washed three times in the same way as mentioned previously. Enzyme-linked antibody was diluted in blocking buffer using a 1:200 ratio and 100 μL was added to each well. The plate was incubated in a 25 ℃ incubator for 2–3 h and washed three times with PBST, as before. Finally, PNPP substrate pills (Agdia Inc., Elkhart, IN, USA) were resolved in substrate buffer (5 mL per pill) and 100 μL was added to each well. The plate was incubated in the dark for 1 h and absorbance values were read using a plate reader at 405 nm.

### 4.6. Small RNA Northern Blot

Total RNA was extracted from top young leaves of potato. miRNA detection was conducted as described previously, with modifications [20]. In brief, a sample of 10 ug was separated by denaturing polyacrylamide gel, transferred to a Hybond N+ membrane (GE Healthcare, Chicago, IL, USA) using the Trans-Blot Turbo (BioRad, Hercules, CA, USA) and crosslinked to the membrane using the CL-1000 Ultraviolet Crosslinker (UVP). Biotin-labeled probes complementary to the four designed amiRs were used in hybridization and then the membrane was processed using a Chemiluminescent Nucleic Acid Detection Module Kit (ThermoFischer, Waltham, MA, USA) according to the manufacture’s protocol, and the signal was visualized using ChemiDoc XRS+ (BioRad).

## Data Availability

Not applicable.

## Supplementary Materials

The following supporting information can be downloaded at: https://www.mdpi.com/article/10.3390/plants11162125/s1, Data S1: Fasta format sequences of artificial miRNA expression vector pAMIR–PLRV derived from pK7LIC1.0; Table S1: Primer sequences used in AMIR synthesis; Table S2: The primers used to construct the miRNA sensor vector PMS4–amiRCP; Figure S1: Sequence alignments of artificial miRNA precursors; Figure S2: Workflow for the graft inoculation on potato plants; Figure S3. Restriction map of vectors used in this study.

## References

[1] Valencia-Sanchez M.A., Liu J., Hannon G.J., Parker R. (2006). Control of translation and mRNA degradation by miRNAs and siRNAs. Genes Dev..

[2] Voinnet O. (2009). Origin, biogenesis, and activity of plant microRNAs. Cell.

[3] Bond D.M., Baulcombe D.C. (2014). Small RNAs and heritable epigenetic variation in plants. Trends Cell Biol..

[4] Rogers K., Chen X. (2013). Biogenesis, turnover, and mode of action of plant microRNAs. Plant Cell.

[5] Axtell M.J. (2013). Classification and comparison of small RNAs from plants. Annu. Rev. Plant Biol..

[6] Deleris A., Gallego-Bartolome J., Bao J., Kasschau K.D., Carrington J.C., Voinnet O. (2006). Hierarchical action and inhibition of plant Dicer-like proteins in antiviral defense. Science.

[7] Diaz-Pendon J.A., Li F., Li W.X., Ding S.W. (2007). Suppression of antiviral silencing by cucumber mosaic virus 2b protein in Arabidopsis is associated with drastically reduced accumulation of three classes of viral small interfering RNAs. Plant Cell.

[8] Wang X.B., Jovel J., Udomporn P., Wang Y., Wu Q., Li W.X., Gasciolli V., Vaucheret H., Ding S.W. (2011). The 21-nucleotide, but not 22-nucleotide, viral secondary small interfering RNAs direct potent antiviral defense by two cooperative argonautes in Arabidopsis thaliana. Plant Cell.

[9] Carbonell A., Carrington J.C. (2015). Antiviral roles of plant ARGONAUTES. Curr. Opin. Plant Biol..

[10] Ding S.W., Voinnet O. (2007). Antiviral immunity directed by small RNAs. Cell.

[11] Guo Z., Li Y., Ding S.W. (2019). Small RNA-based antimicrobial immunity. Nat. Rev. Immunol..

[12] Li F., Ding S.W. (2006). Virus counterdefense: Diverse strategies for evading the RNA-silencing immunity. Annu. Rev. Microbiol..

[13] Burgyan J., Havelda Z. (2011). Viral suppressors of RNA silencing. Trends Plant Sci..

[14] Zhao S., Wu Y., Wu J. (2021). Arms race between rice and viruses: A review of viral and host factors. Curr. Opin. Virol..

[15] Khalid A., Zhang Q., Yasir M., Li F. (2017). Small RNA Based Genetic Engineering for Plant Viral Resistance: Application in Crop Protection. Front. Microbiol..

[16] Schwab R., Ossowski S., Riester M., Warthmann N., Weigel D. (2006). Highly specific gene silencing by artificial microRNAs in Arabidopsis. Plant Cell.

[17] Niu Q.W., Lin S.S., Reyes J.L., Chen K.C., Wu H.W., Yeh S.D., Chua N.H. (2006). Expression of artificial microRNAs in transgenic Arabidopsis thaliana confers virus resistance. Nat. Biotechnol..

[18] Liu Y., Li F., Li Y., Zhang S., Gao X., Xie Y., Yan F., Zhang A., Dai L., Cheng Z. (2019). Identification, Distribution and Occurrence of Viruses in the Main Vegetables of China. Sci. Agric. Sin..

[19] Lu C., Kulkarni K., Souret F.F., MuthuValliappan R., Tej S.S., Poethig R.S., Henderson I.R., Jacobsen S.E., Wang W., Green P.J. (2006). MicroRNAs and other small RNAs enriched in the Arabidopsis RNA-dependent RNA polymerase-2 mutant. Genome Res..

[20] Li F., Pignatta D., Bendix C., Brunkard J.O., Cohn M.M., Tung J., Sun H., Kumar P., Baker B. (2012). MicroRNA regulation of plant innate immune receptors. Proc. Natl. Acad. Sci. USA.

[21] Chen H.M., Chen L.T., Patel K., Li Y.H., Baulcombe D.C., Wu S.H. (2010). 22-Nucleotide RNAs trigger secondary siRNA biogenesis in plants. Proc. Natl. Acad. Sci. USA.

[22] Cuperus J.T., Carbonell A., Fahlgren N., Garcia-Ruiz H., Burke R.T., Takeda A., Sullivan C.M., Gilbert S.D., Montgomery T.A., Carrington J.C. (2010). Unique functionality of 22-nt miRNAs in triggering RDR6-dependent siRNA biogenesis from target transcripts in Arabidopsis. Nat. Struct. Mol. Biol..

[23] McHale M., Eamens A.L., Finnegan E.J., Waterhouse P.M. (2013). A 22-nt artificial microRNA mediates widespread RNA silencing in Arabidopsis. Plant J..

[24] Ossowski S., Schwab R., Weigel D. (2008). Gene silencing in plants using artificial microRNAs and other small RNAs. Plant J..

[25] Fahlgren N., Hill S.T., Carrington J.C., Carbonell A. (2016). P-SAMS: A web site for plant artificial microRNA and synthetic trans-acting small interfering RNA design. Bioinformatics.

[26] Zhang H., Feng H., Lu X., Wang C., Yang W., Li F. (2020). An asymmetric bulge enhances artificial microRNA-mediated virus resistance. Plant Biotechnol. J..

[27] Liu T., Fang H., Liu J., Reid S., Hou J., Zhou T., Tian Z., Song B., Xie C. (2017). Cytosolic glyceraldehyde-3-phosphate dehydrogenases play crucial roles in controlling cold-induced sweetening and apical dominance of potato (*Solanum tuberosum* L.) tubers. Plant Cell Environ..

[28] Wang J., Meng F., Chen R., Liu J., Nie X., Nie B. (2016). RT-PCR Differentiation, Molecular and Pathological Characterization of Andean and Ordinary Strains of Potato virus S in Potatoes in China. Plant Dis..

