# Supplementary material for: Simple Webserver-Facilitated Method to Design and Synthesize Artificial miRNA Gene and Its Application in Engineering Viral Resistance
Source: Plants (Basel). 2022 Aug 15;11(16):2125. doi: 10.3390/plants11162125 (PMC9412884; doi:10.3390/plants11162125)
Supplement: Supplementary file 1 [file plants-11-02125-s001.zip › plants-1789959-SM.pdf]

# Supplementary Materials

**Data S1. Fasta format sequences of artificial miRNA expression vector pAMIR–PLRV derived from pK7LIC1.0**.

>**pAMIR-PLRV**

GGTACCCTCGAATTATCATACATGAGAATTAAGGGAGTCACGTTATGACCCCCGCCGATGACGCGGGACA
AGCCGTTTTACGTTTGGAACTGACAGAACCGCAACGTTGAAGGAGCCACTCAGCCGCGGGTTTCTGGAGT
TTAATGAGCTAAGCACATACGTCAGAAACCATTATTGCGCGTTCAAAAGTCGCCTAAGGTCACTATCAGC
TAGCAAATATTTCTTGTCAAAAATGCTCCACTGACGTTCCATAAATTCCCCTCGGTATCCAATTAGAGTC
TCATATTCACTCTCAACTCGATCGAGGCATGATTGAACAAGATGGATTGCACGCAGGTTCTCCGGCCGCT
TGGGTGGAGAGGCTATTCGGCTATGACTGGGCACAACAGACAATCGGCTGCTCTGATGCCGCCGTGTTCC
GGCTGTCAGCGCAGGGGCGCCCGGTTCTTTTTGTCAAGACCGACCTGTCCGGTGCCCTGAATGAACTCCA
AGACGAGGCAGCGCGGCTATCGTGGCTGGCCACGACGGGCGTTCCTTGCGCAGCTGTGCTCGACGTTGTC
ACTGAAGCGGGAAGGGACTGGCTGCTATTGGGCGAAGTGCCGGGGCAGGATCTCCTGTCATCTCACCTTG
CTCCTGCCGAGAAAGTATCCATCATGGCTGATGCAATGCGGCGGCTGCATACGCTTGATCCGGCTACCTG
CCCATTCGACCACCAAGCGAAACATCGCATCGAGCGAGCACGTACTCGGATGGAAGCCGGTCTTGTCGAT
CAGGATGATCTGGACGAAGAGCATCAGGGGCTCGCGCCAGCCGAACTGTTCGCCAGGCTCAAGGCGCGGA
TGCCCGACGGCGAGGATCTCGTCGTGACCCACGGCGATGCCTGCTTGCCGAATATCATGGTGGAAAATGG
CCGCTTTTCTGGATTCATCGACTGTGGCCGGCTGGGTGTGGCGGACCGCTATCAGGACATAGCGTTGGCT
ACCCGTGATATTGCTGAAGAGCTTGGCGGCGAATGGGCTGACCGCTTCCTCGTGCTTTACGGTATCGCCG
CTCCCGATTCGCAGCGCATCGCCTTCTATCGCCTTCTTGACGAGTTCTTCTGAGCGGGACTCTGGGGTTC
GGACTCTAGCTAGAGTCAAGCAGATCGTTCAAACATTTGGCAATAAAGTTTCTTAAGATTGAATCCTGTT
GCCGGTCTTGCGATGATTATCATATAATTTCTGTTGAATTACGTTAAGCATGTAATAATTAACATGTAAT
GCATGACGTTATTTATGAGATGGGTTTTTATGATTAGAGTCCCGCAATTATACATTTAATACGCGATAGA
AAACAAAATATAGCGCGCAAACTAGGATAAATTATCGCGCGCGGTGTCATCTATGTTACTAGATCGACCG
GCATGCAAGCTGATAATTCAATTCGGCGTTAATTCAGTACATTAAAAACGTCCGCAATGTGTTATTAAGT
TGTCTAAGCGTCAATTTGTTTACACCACAATATATCCTGCCACCAGCCAGCCAACAGCTCCCCGACCGGC
AGCTCGGCACAAAATCACCACTCGATACAGGCAGCCCATCAGTCCGGGACGGCGTCAGCGGGAGAGCCGT
TGTAAGGCGGCAGACTTTGCTCATGTTACCGATGCTATTCGGAAGAACGGCAACTAAGCTGCCGGGTTTG
AAACACGGATGATCTCGCGGAGGGTAGCATGTTGATTGTAACGATGACAGAGCGTTGCTGCCTGTGATCA
ATTCGGGCACGAACCCAGTGGACATAAGCCTCGTTCGGTTCGTAAGCTGTAATGCAAGTAGCGTAACTGC
CGTCACGCAACTGGTCCAGAACCTTGACCGAACGCAGCGGTGGTAACGGCGCAGTGGCGGTTTTCATGGC
TTCTTGTTATGACATGTTTTTTTGGGGTACAGTCTATGCCTCGGGCATCCAAGCAGCAAGCGCGTTACGC
CGTGGGTCGATGTTTGATGTTATGGAGCAGCAACGATGTTACGCAGCAGGGCAGTCGCCCTAAAACAAAG
TTAAACATCATGGGGGAAGCGGTGATCGCCGAAGTATCGACTCAACTATCAGAGGTAGTTGGCGTCATCG
AGCGCCATCTCGAACCGACGTTGCTGGCCGTACATTTGTACGGCTCCGCAGTGGATGGCGGCCTGAAGCC
ACACAGTGATATTGATTTGCTGGTTACGGTGACCGTAAGGCTTGATGAAACAACGCGGCGAGCTTTGATC
AACGACCTTTTGGAAACTTCGGCTTCCCCTGGAGAGAGCGAGATTCTCCGCGCTGTAGAAGTCACCATTG
TTGTGCACGACGACATCATTCCGTGGCGTTATCCAGCTAAGCGCGAACTGCAATTTGGAGAATGGCAGCG
CAATGACATTCTTGCAGGTATCTTCGAGCCAGCCACGATCGACATTGATCTGGCTATCTTGCTGACAAAA
GCAAGAGAACATAGCGTTGCCTTGGTAGGTCCAGCGGCGGAGGAACTCTTTGATCCGGTTCCTGAACAGG
ATCTATTTGAGGCGCTAAATGAAACCTTAACGCTATGGAACTCGCCGCCCGACTGGGCTGGCGATGAGCG

AAATGTAGTGCTTACGTTGTCCCGCATTTGGTACAGCGCAGTAACCGGCAAAATCGCGCCGAAGGATGTC
GCTGCCGACTGGGCAATGGAGCGCCTGCCGGCCCAGTATCAGCCCGTCATACTTGAAGCTAGACAGGCTT
ATCTTGGACAAGAAGAAGATCGCTTGGCCTCGCGCGCAGATCAGTTGGAAGAATTTGTCCACTACGTGAA
AGGCGAGATCACCAAGGTAGTCGGCAAATAATGTCTAGCTAGAAATTCGTTCAAGCCGACGCCGCTTCGC
CGGCGTTAACTCAAGCGATTAGATGCACTAAGCACATAATTGCTCACAGCCAAACTATCAGGTCAAGTCT
GCTTTTATTATTTTTAAGCGTGCATAATAAGCCCTACACAAATTGGGAGATATATCATGCATGACCAAAA
TCCCTTAACGTGAGTTTTCGTTCCACTGAGCGTCAGACCCCGTAGAAAAGATCAAAGGATCTTCTTGAGA
TCCTTTTTTTCTGCGCGTAATCTGCTGCTTGCAAACAAAAAAACCACCGCTACCAGCGGTGGTTTGTTTG
CCGGATCAAGAGCTACCAACTCTTTTTCCGAAGGTAACTGGCTTCAGCAGAGCGCAGATACCAAATACTG
TCCTTCTAGTGTAGCCGTAGTTAGGCCACCACTTCAAGAACTCTGTAGCACCGCCTACATACCTCGCTCT
GCTAATCCTGTTACCAGTGGCTGCTGCCAGTGGCGATAAGTCGTGTCTTACCGGGTTGGACTCAAGACGA
TAGTTACCGGATAAGGCGCAGCGGTCGGGCTGAACGGGGGGTTCGTGCACACAGCCCAGCTTGGAGCGAA
CGACCTACACCGAACTGAGATACCTACAGCGTGAGCTATGAGAAAGCGCCACGCTTCCCGAAGGGAGAAA
GGCGGACAGGTATCCGGTAAGCGGCAGGGTCGGAACAGGAGAGCGCACGAGGGAGCTTCCAGGGGGAAAC
GCCTGGTATCTTTATAGTCCTGTCGGGTTTCGCCACCTCTGACTTGAGCGTCGATTTTTGTGATGCTCGT
CAGGGGGGCGGAGCCTATGGAAAAACGCCAGCAACGCGGCCTTTTTACGGTTCCTGGCCTTTTGCTGGCC
TTTTGCTCACATGTTCTTTCCTGCGTTATCCCCTGATTCTGTGGATAACCGTATTACCGCCTTTGAGTGA
GCTGATACCGCTCGCCGCAGCCGAACGACCGAGCGCAGCGAGTCAGTGAGCGAGGAAGCGGAAGAGCGCC
TGATGCGGTATTTTCTCCTTACGCATCTGTGCGGTATTTCACACCGCATATGGTGCACTCTCAGTACAAT
CTGCTCTGATGCCGCATAGTTAAGCCAGTATACACTCCGCTATCGCTACGTGACTGGGTCATGGCTGCGC
CCCGACACCCGCCAACACCCGCTGACGCGCCCTGACGGGCTTGTCTGCTCCCGGCATCCGCTTACAGACA
AGCTGTGACCGTCTCCGGGAGCTGCATGTGTCAGAGGTTTTCACCGTCATCACCGAAACGCGCGAGGCAG
GGTGCCTTGATGTGGGCGCCGGCGGTCGAGTGGCGACGGCGCGGCTTGTCCGCGCCCTGGTAGATTGCCT
GGCCGTAGGCCAGCCATTTTTGAGCGGCCAGCGGCCGCGATAGGCCGACGCGAAGCGGCGGGGCGTAGGG
AGCGCAGCGACCGAAGGGTAGGCGCTTTTTGCAGCTCTTCGGCTGTGCGCTGGCCAGACAGTTATGCACA
GGCCAGGCGGGTTTTAAGAGTTTTAATAAGTTTTAAAGAGTTTTAGGCGGAAAAATCGCCTTTTTTCTCT
TTTATATCAGTCACTTACATGTGTGACCGGTTCCCAATGTACGGCTTTGGGTTCCCAATGTACGGGTTCC
GGTTCCCAATGTACGGCTTTGGGTTCCCAATGTACGTGCTATCCACAGGAAAGAGACCTTTTCGACCTTT
TTCCCCTGCTAGGGCAATTTGCCCTAGCATCTGCTCCGTACATTAGGAACCGGCGGATGCTTCGCCCTCG
ATCAGGTTGCGGTAGCGCATGACTAGGATCGGGCCAGCCTGCCCCGCCTCCTCCTTCAAATCGTACTCCG
GCAGGTCATTTGACCCGATCAGCTTGCGCACGGTGAAACAGAACTTCTTGAACTCTCCGGCGCTGCCACT
GCGTTCGTAGATCGTCTTGAACAACCATCTGGCTTCTGCCTTGCCTGCGGCGCGGCGTGCCAGGCGGTAG
AGAAAACGGCCGATGCCGGGATCGATCAAAAAGTAATCGGGGTGAACCGTCAGCACGTCCGGGTTCTTGC
CTTCTGTGATCTCGCGGTACATCCAATCAGCTAGCTCGATCTCGATGTACTCCGGCCGCCCGGTTTCGCT
CTTTACGATCTTGTAGCGGCTAATCAAGGCTTCACCCTCGGATACCGTCACCAGGCGGCCGTTCTTGGCC
TTCTTCGTACGCTGCATGGCAACGTGCGTGGTGTTTAACCGAATGCAGGTTTCTACCAGGTCGTCTTTCT
GCTTTCCGCCATCGGCTCGCCGGCAGAACTTGAGTACGTCCGCAACGTGTGGACGGAACACGCGGCCGGG
CTTGTCTCCCTTCCCTTCCCGGTATCGGTTCATGGATTCGGTTAGATGGGAAACCGCCATCAGTACCAGG
TCGTAATCCCACACACTGGCCATGCCGGCCGGCCCTGCGGAAACCTCTACGTGCCCGTCTGGAAGCTCGT
AGCGGATCACCTCGCCAGCTCGTCGGTCACGCTTCGACAGACGGAAAACGGCCACGTCCATGATGCTGCG
ACTATCGCGGGTGCCCACGTCATAGAGCATCGGAACGAAAAAATCTGGTTGCTCGTCGCCCTTGGGCGGC
TTCCTAATCGACGGCGCACCGGCTGCCGGCGGTTGCCGGGATTCTTTGCGGATTCGATCAGCGGCCGCTT

GCCACGATTCACCGGGGCGTGCTTCTGCCTCGATGCGTTGCCGCTGGGCGGCCTGCGCGGCCTTCAACTT
CTCCACCAGGTCATCACCCAGCGCCGCGCCGATTTGTACCGGGCCGGATGGTTTGCGACCGTCACGCCGA
TTCCTCGGGCTTGGGGGTTCCAGTGCCATTGCAGGGCCGGCAGACAACCCAGCCGCTTACGCCTGGCCAA
CCGCCCGTTCCTCCACACATGGGGCATTCCACGGCGTCGGTGCCTGGTTGTTCTTGATTTTCCATGCCGC
CTCCTTTAGCCGCTAAAATTCATCTACTCATTTATTCATTTGCTCATTTACTCTGGTAGCTGCGCGATGT
ATTCAGATAGCAGCTCGGTAATGGTCTTGCCTTGGCGTACCGCGTACATCTTCAGCTTGGTGTGATCCTC
CGCCGGCAACTGAAAGTTGACCCGCTTCATGGCTGGCGTGTCTGCCAGGCTGGCCAACGTTGCAGCCTTG
CTGCTGCGTGCGCTCGGACGGCCGGCACTTAGCGTGTTTGTGCTTTTGCTCATTTTCTCTTTACCTCATT
AACTCAAATGAGTTTTGATTTAATTTCAGCGGCCAGCGCCTGGACCTCGCGGGCAGCGTCGCCCTCGGGT
TCTGATTCAAGAACGGTTGTGCCGGCGGCGGCAGTGCCTGGGTAGCTCACGCGCTGCGTGATACGGGACT
CAAGAATGGGCAGCTCGTACCCGGCCAGCGCCTCGGCAACCTCACCGCCGATGCGCGTGCCTTTGATCGC
CCGCGACACGACAAAGGCCGCTTGTAGCCTTCCATCCGTGACCTCAATGCGCTGCTTAACCAGCTCCACC
AGGTCGGCGGTGGCCCATATGTCGTAAGGGCTTGGCTGCACCGGAATCAGCACGAAGTCGGCTGCCTTGA
TCGCGGACACAGCCAAGTCCGCCGCCTGGGGCGCTCCGTCGATCACTACGAAGTCGCGCCGGCCGATGGC
CTTCACGTCGCGGTCAATCGTCGGGCGGTCGATGCCGACAACGGTTAGCGGTTGATCTTCCCGCACGGCC
GCCCAATCGCGGGCACTGCCCTGGGGATCGGAATCGACTAACAGAACATCGGCCCCGGCGAGTTGCAGGG
CGCGGGCTAGATGGGTTGCGATGGTCGTCTTGCCTGACCCGCCTTTCTGGTTAAGTACAGCGATAACCTT
CATGCGTTCCCCTTGCGTATTTGTTTATTTACTCATCGCATCATATACGCAGCGACCGCATGACGCAAGC
TGTTTTACTCAAATACACATCACCTTTTTAGACGGCGGCGCTCGGTTTCTTCAGCGGCCAAGCTGGCCGG
CCAGGCCGCCAGCTTGGCATCAGACAAACCGGCCAGGATTTCATGCAGCCGCACGGTTGAGACGTGCGCG
GGCGGCTCGAACACGTACCCGGCCGCGATCATCTCCGCCTCGATCTCTTCGGTAATGAAAAACGGTTCGT
CCTGGCCGTCCTGGTGCGGTTTCATGCTTGTTCCTCTTGGCGTTCATTCTCGGCGGCCGCCAGGGCGTCG
GCCTCGGTCAATGCGTCCTCACGGAAGGCACCGCGCCGCCTGGCCTCGGTGGGCGTCACTTCCTCGCTGC
GCTCAAGTGCGCGGTACAGGGTCGAGCGATGCACGCCAAGCAGTGCAGCCGCCTCTTTCACGGTGCGGCC
TTCCTGGTCGATCAGCTCGCGGGCGTGCGCGATCTGTGCCGGGGTGAGGGTAGGGCGGGGGCCAAACTTC
ACGCCTCGGGCCTTGGCGGCCTCGCGCCCGCTCCGGGTGCGGTCGATGATTAGGGAACGCTCGAACTCGG
CAATGCCGGCGAACACGGTCAACACCATGCGGCCGGCCGGCGTGGTGGTGTCGGCCCACGGCTCTGCCAG
GCTACGCAGGCCCGCGCCGGCCTCCTGGATGCGCTCGGCAATGTCCAGTAGGTCGCGGGTGCTGCGGGCC
AGGCGGTCTAGCCTGGTCACTGTCACAACGTCGCCAGGGCGTAGGTGGTCAAGCATCCTGGCCAGCTCCG
GGCGGTCGCGCCTGGTGCCGGTGATCTTCTCGGAAAACAGCTTGGTGCAGCCGGCCGCGTGCAGTTCGGC
CCGTTGGTTGGTCAAGTCCTGGTCGTCGGTGCTGACGCGGGCATAGCCCAGCAGGCCAGCGGCGGCGCTC
TTGTTCATGGCGTAATGTCTCCGGTTCTAGTCGCAAGTATTCTACTTTATGCGACTAAAACACGCGACAA
GAAAACGCCAGGAAAAGGGCAGGGCGGCAGCCTGTCGCGTAACTTAGGACTTGTGCGACATGTCGTTTTC
AGAAGACGGCTGCACTGAACGTCAGAAGCCGACTGCACTATAGCAGCGGAGGGGTTGGATCAAAGTACTT
TGATCCCGAGGGGAACCCTGTGGTTGGCATGCACATACAAATGGACGAACGGATAAACCTTTTCACGCCC
TTTTAAATATCCGTTATTCTAATAAACGCTCTTTTCTCTTAGGTTTACCCGCCAATATATCCTGTCAAAC
ACTGATAGTTTAAACTGAAGGCGGGAAACGACAATCTGATCCAAGCTCAAGCTAAGCTTGAGCTCTCCCA
TATGGTCGACTAGAGCCAAGCTGATCTCCTTTGCCCCGGAGATCACCATGGACGACTTTCTCTATCTCTA
CGATCTAGGAAGAAAGTTCGACGGAGAAGGTGACGATACCATGTTCACCACCGATAATGAGAAGATTAGC
CTCTTCAATTTCAGAAAGAATGCTGACCCACAGATGGTTAGAGAGGCCTACGCGGCAGGTCTCATCAAGA
CGATCTACCCGAGTAATAATCTCCAGGAGATCAAATACCTTCCCAAGAAGGTTAAAGATGCAGTCAAAAG
ATTCAGGACTAACTGCATCAAGAACACAGAGAAAGATATATTTCTCAAGATCAGAAGTACTATTCCAGTA

TGGACGATTCAAGGCTTGCTTCATAAACCAAGGCAAGTAATAGAGATTGGAGTCTCTAAGAAAGTAGTTC
CTACTGAATCAAAGGCCATGGAGTCAAAAATTCAGATCGAGGATCTAACAGAACTCGCCGTGAAGACTGG
CGAACAGTTCATACAGAGTCTTTTACGACTCAATGACAAGAAGAAAATCTTCGTCAACATGGTGGAGCAC
GACACTCTCGTCTACTCCAAGAATATCAAAGATACAGTCTCAGAAGACCAAAGGGCTATTGAGACTTTTC
AACAAAGGGTAATATCGGGAAACCTCCTCGGATTCCATTGCCCAGCTATCTGTCACTTCATCAAAAGGAC
AGTAGAAAAGGAAGGTGGCACCTACAAATGCCATCATTGCGATAAAGGAAAGGCTATCGTTCAAGATGCC
TCTGCCGACAGTGGTCCCAAAGATGGACCCCCACCCACGAGGAGCATCGTGGAAAAAGAAGACGTTCCAA
CCACGTCTTCAAAGCAAGTGGATTGATGTGATGATATCATCTCCACTGACGTAAGGGATGACGCACAATC
CCACTATCCTTCGCAAGACCCTTCCTCTATATAAGGAAGTTCATTTCATTTGGAGAGGACTCCGGTATTT
TTACAACAATACCACAACAAAACAAACAACAAACAACATTACAATTTACTATTCTAGTCGACCTGCAGGC
GGCCGCAACAACTAGTCCAGGGCGCCCTCACCAAACAAAACCTCACAATGAGAGAGTCCCTGTTCTGGAT
TGCGGATGAGAATCCTTTCTTACCTGACCACACACGTAGATATACATTATTCTCTCATGATTAAAGGATT
CTCCTCCGCAACCCAGATCAGTACTCTCTCGTCAACCAAAGTAAGTAATCACACATAAGAGCTTGGCGGC
GAATCACCAAACCTCCACCAACAATGGGTGAGATTCTCCATTCTCTTGAATGCCGGACAGTCTGTAACCA
ACAAACACGAAATCCGTCACATTTGCTTATTACAGACTGTCAGGCATTCCAGAGAATGAGCACTTCACCC
CAATACCATTACCCAACAGAACTCTGAGCACCCAGTCCGCCCACCACCGTCACAGCCAAATACACAGAGT
CCCTGTTCTCTGGTTATACAACCGAGCAATTCACTTCTGACCACACACAAGTATTATATATACAGTCTCT
ACTTTAATTGCTCGGTCGTATAACAAGAGATCAGTACTCTGTGTAACACAAACCGCAAACATTCTACCCA
GCCACAACGTCTATATCCACCACACCCACAAACTACTCGGCACAGAATGACGATTCTCAAGCTGGTTATA
CAACCGATAACCAAAACTCACGAAATCCGTCTACAGTTCTTATTATCGGTTGTACAACCAGCCTGAGAAT
CTCCTCTTGTGCCAAACCATCAATCCCAACTAATCAAGGGCGTCGGGTCGAGTGGCAGTGAAGGGCGAAC
AGTTCCTGATTAACCACAAACCGTTCTACTTTACTGGCTTTGGTCGTCATGAAGATGCGGACTTGCGTGG
CAAAGGATTCGATAACGTGCTGATGGTGCACGACCACGCATTAATGGACTGGATTGGGGCCAACTCCTAC
CGTACCTCGCATTACCCTTACGCTGAAGAGATGCTCGACTTCTAGAGCACTCGAGATTGGCGTCGGGTCG
ATGATATCCCGCGGCCATGCTAGAGTCCGCAAAAATCACCAGTCTCTCTCTACAAATCTATCTCTCTCTA
TTTTTCTCCAGAATAATGTGTGAGTAGTTCCCAGATAAGGGAATTAGGGTTCTTATAGGGTTTCGCTCAT
GTGTTGAGCATATAAGAAACCCTTAGTATGTATTTGTATTTGTAAAATACTTCTATCAATAAAATTTCTA
ATTCCTAAAACCAAAATCCAGTGACCTGCAGGCATGCGACGTCGG

* Sequences with a yellow background are AMIR sequences and those with a blue background are connector sequences.

**Table S1. Primer sequences used in AMIR synthesis.**

| Gene ID (backbone) | Primer ID | Primer Sequences |
|---|---|---|
| AMIR3a (AMIR171v1) | YRPD0001 | ACA**ACTAGT**CCAGGGCGCCCTCACCAAACAAAACCTCACAATGAGAGAGTCCCTGT<br>SpeI |
| | YRPD0107 | GTGTGTGGTCAGGTAAGAAAGGATTCTCATCCGCAATCCAGAACAGGGACTCTCTCA |
| | YRPD0003 | TTCTTACCTGACCACACACGTAGATATACATTATTCTCTCATGATTAA |
| | YRPD0108 | ATTATTCTCTCATGATTAAAGGATTCTCCTCCGCAACCCAGATCAGTACTCTCTCGTCAA |
| | YRPD0005 | ATTCGCCGCCAAGCTCTTATGTGTGATTACTTACTTTGGTTGACGAGAGAGTACTGA |
| AMIRCP (AMIR164v1) | YRPD0006 | AAGAGCTTGGCGGCGAATCACCAAACCTCCACCAACAATGGGTGAGATTCTCCAT |
| | YRPD0109 | ATTTCGTGTTTGTTGGTTACAGACTGTCCGGCATTCAAGAGAATGGAGAATCTCACC |
| | YRPD0008 | AACCAACAAACACGAAATCCGTCACATTTGCTTATT |
| | YRPD0110 | CCGTCACATTTGCTTATTACAGACTGTCAGGCATTCCAGAGAATGAGCACTTCACCCCAA |
| | YRPD0010 | GGCGGACTGGGTGCTCAGAGTTCTGTTGGGTAATGGTATTGGGGTGAAGTGCTCAT |
| AMIRP1 (AMIR171v2) | YRPD0011 | CTGAGCACCCAGTCCGCCCACCACCGTCACAGCCAAATACACAGAGTCCCTGT |
| | YRPD0111 | GTGTGTGGTCAGAAGTGAATTGCTCGGTTGTATAACCAGAGAACAGGGACTCTGTGT |
| | YRPD013 | TCACTTCTGACCACACACAAGTATTATATATACAGTCTCTACTTTA |
| | YRPD0112 | TATACAGTCTCTACTTTAATTGCTCGGTCGTATAACAAGAGATCAGTACTCTGTGTAACA |
| | YRPD0015 | GATATAGACGTTGTGGCTGGGTAGAATGTTTGCGGTTTGTGTTACACAGAGTACTGA |
| AMIRP0 (AMIR164v2) | YRPD0016 | AGCCACAACGTCTATATCCACCACACCCACAAACTACTCGGCACAGAATGACGAT |
| | YRPD0113 | ATTTCGTGAGTTTTGGTTATCGGTTGTATAACCAGCTTGAGAATCGTCATTCTGTGC |
| | YRPD0018 | AACCAAAACTCACGAAATCCGTCTACAGTTCTTATT |
| | YRPD0114 | CCGTCTACAGTTCTTATTATCGGTTGTACAACCAGCCTGAGAATCTCCTCTTGTGCCAAA |
| | YRPD0020 | TGATTAGTTGGGATTGATGGTTTGGCACAAGAGGAGAT |
| GUS4 | YRPD0051 | ATCAATCCCAACTAATCAAGGGCGTCGGGTCGAGTGGCAGTGAAGGGCGAAC |
| | YRPD0052 | AAT**CTCGAG**TGC**TCTAGA**AGTCGAGCATCTCTTCAGCG<br>XhoI XbaI |

Sequences with a yellow background are primer sequences designed in AMIRdesigner; sequences with a blue background are connector sequences added for cluster AMIR and GUS sequences by fusion PCR; sequences in bold and underlined encode restriction sites, as indicated below the lines.

## Table S2 The primers used to construct miRNA sensor vector PMS4–amiRCP.

| Primer ID | Primer sequence (5′-3′) |
|---|---|
| Anti-AMIRCP_F | **TCGA**GTCTCAGACTGTCCGGCATTCAAGAT |
| Anti-AMIRCP_R | **CTAG**ATCTTGAATGCCGGACAGTCTGAGAC |

Letters in bold blue form a 5’ overhang compatible with XhoI and XbaI digested vector DNA; underlined sequences are complementary in the F and R primers; letters of the F primer and R primer in red encode the amiRCP binding site and its complementary sequences, respectively.

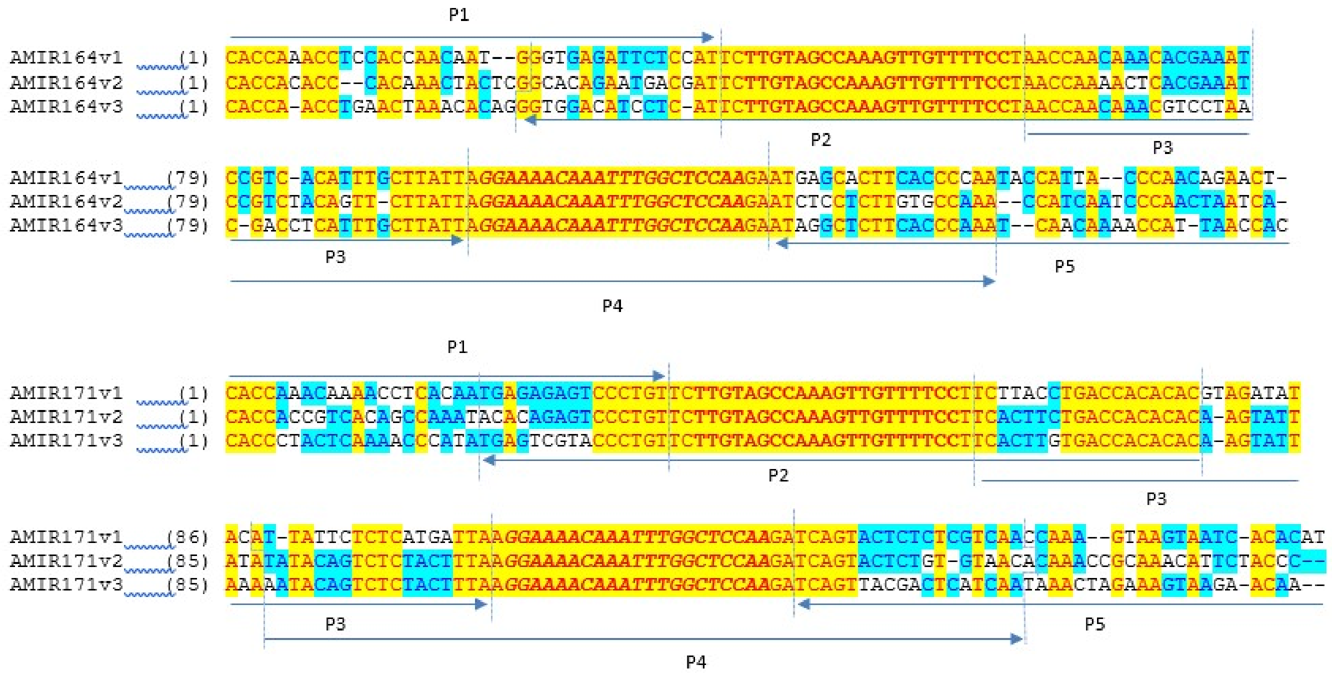


**Figure S1 Sequence alignments of artificial miRNA precursors.** Arrows represent primers P1 to P5, with forward primers pointing to the right and reverse primers pointing to the left. Dashed lines indicate the ends of each primers.

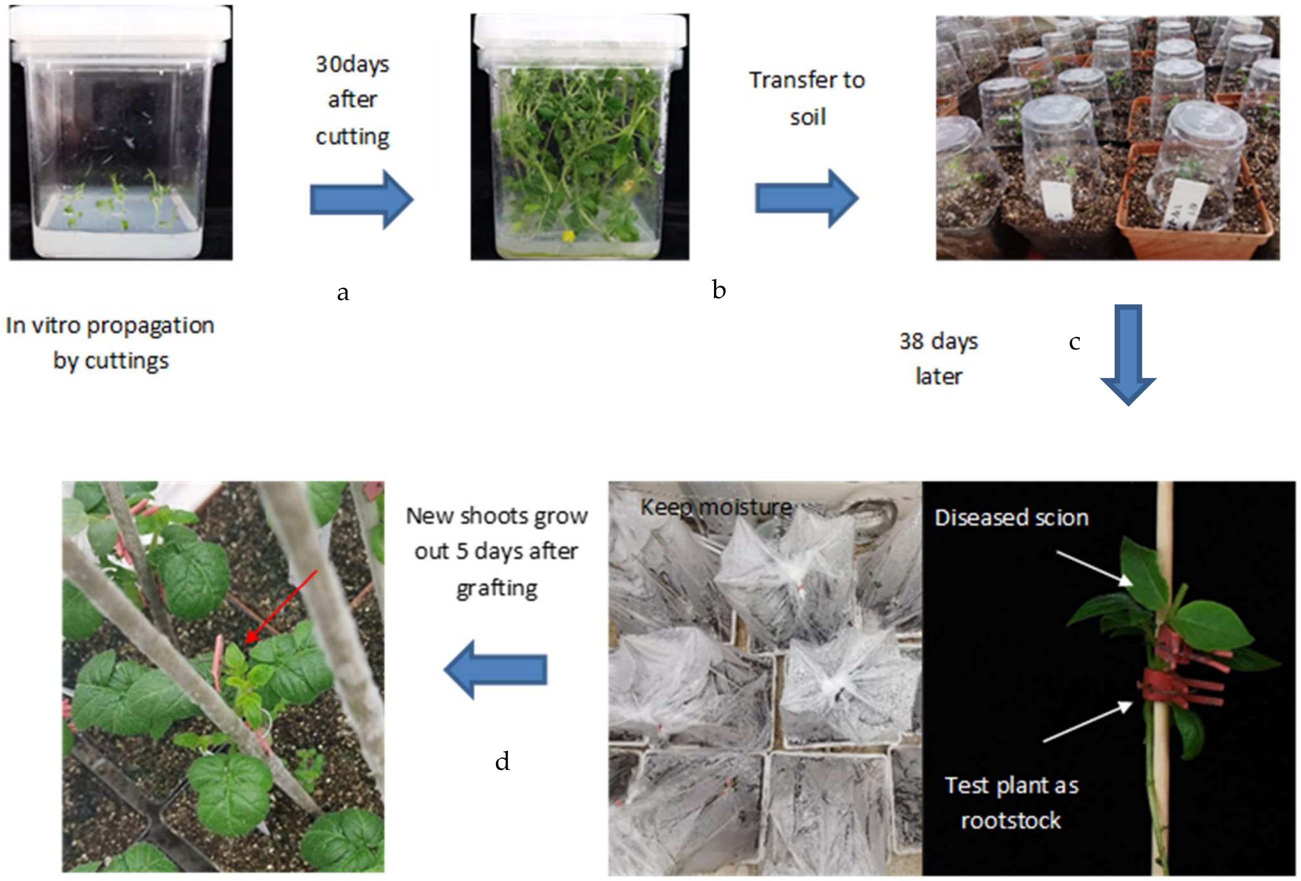


**Figure S2. Workflow for graft inoculation on potato plants.** a) Grow potato seedlings from cutting for 30 days in tissue culture medium; b) transfer potato seedlings to soil; c) 38 days later, transfer potato seedlings to soil, graft diseased scion onto the potato seedlings and retain moisture using plastic cover; d) continue to grow grafted potato plants to allow new shoots to grow.

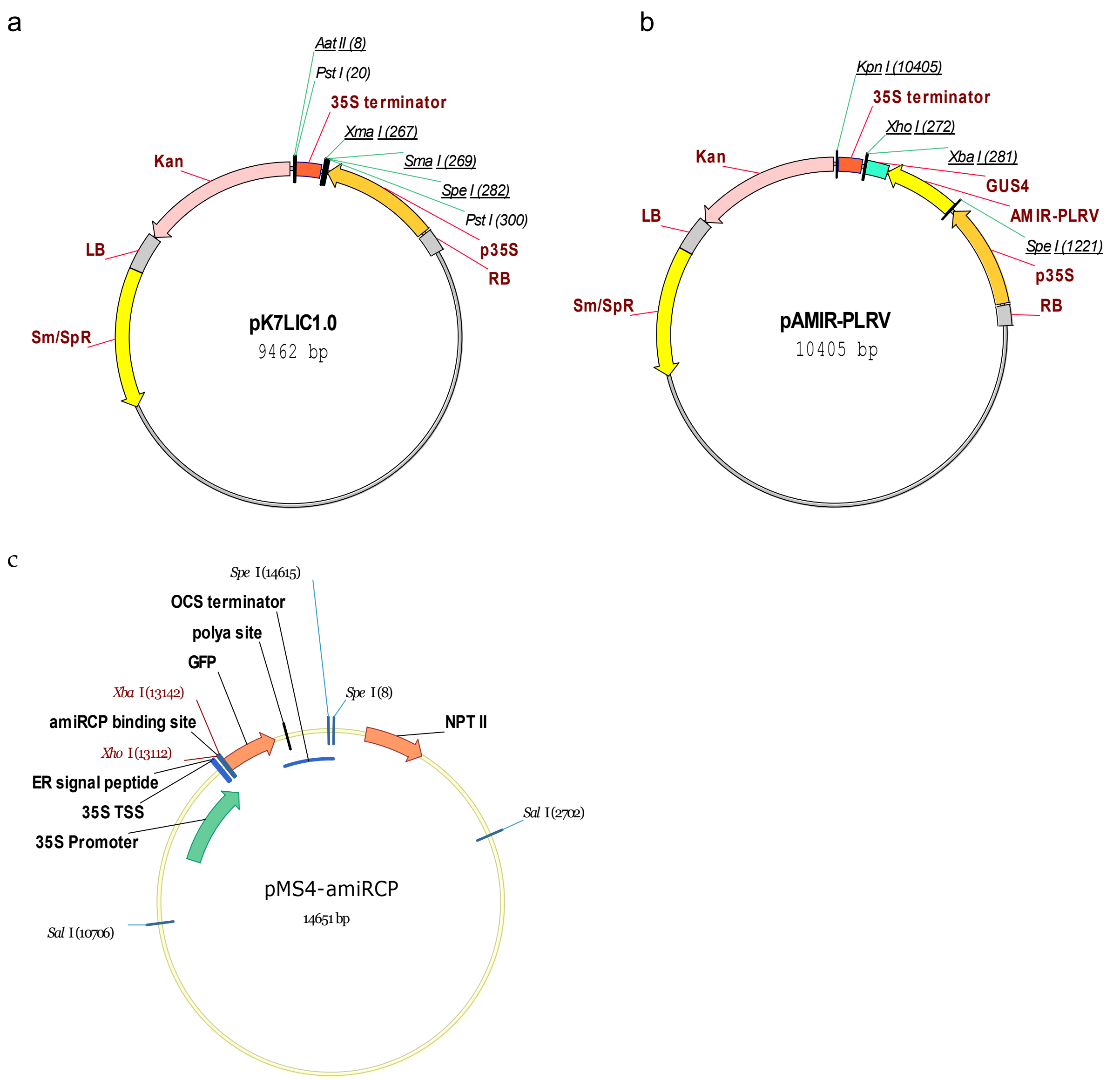


**Figure S3. Restriction maps of vectors used in this study.** a) Vector pK7LIC1.0 was derived from gateway vector pK7WGF2 (VIB—UGent Center for Plant Systems Biology ), replacing its LR recombination unit and eGFP with ligation-independent cloning (LIC) sequences. The LIC sequences are split by a SmaI site. SpeI and PstI sites are placed next to the LIC sequences. b) pAMIR–PLRV. The AMIR–PLRV cluster and GUS spacer sequences are shown as yellow arrows and a blue square between the 35S terminator (red square) and 35S promoter (dark yellow arrow). c) pMS4–amiRCP. The amiRCP binding site is flanked by XhoI and XbaI restriction sites.
